# Why Industry Propaganda and Political Interference Cannot Disguise the Inevitable Role Played by Human Exposure to Aluminum in Neurodegenerative Diseases, Including Alzheimer’s Disease

**DOI:** 10.3389/fneur.2014.00212

**Published:** 2014-10-27

**Authors:** Christopher Exley

**Affiliations:** ^1^The Birchall Centre, Lennard-Jones Laboratories, Keele University, Stoke-on-Trent, UK

**Keywords:** aluminium, Alzheimer’s disease, human exposure, neurodegenerative disease, body burden

## Abstract

In the aluminum age, it is clearly unpalatable for aluminum, the globe’s most successful metal, to be implicated in human disease. It is unpalatable because for approximately 100 years human beings have reaped the rewards of the most abundant metal of the Earth’s crust without seriously considering the potential consequences for human health. The aluminum industry is a pillar of the developed and developing world and irrespective of the tyranny of human exposure to aluminum it cannot be challenged without significant consequences for businesses, economies, and governments. However, no matter how deep the dependency or unthinkable the withdrawal, science continues to document, if not too slowly, a burgeoning body burden of aluminum in human beings. Herein, I will make the case that it is inevitable both today and in the future that an individual’s exposure to aluminum is impacting upon their health and is already contributing to, if not causing, chronic diseases such as Alzheimer’s disease. This is the logical, if uncomfortable, consequence of living in the aluminum age.

## Introduction

In 1970, it was believed that the phosphorylation of glucose by hexokinase in the presence of ATP required certain “activators,” such as citrate, and especially so at circumneutral pH (1). In 1979, the so-called allosteric activity of citrate (and other activators) was found to be the consequence of the contamination of laboratory supplies of ATP by aluminum (2). The same problems of contamination are an issue today (3, 4) and though the level of contamination is usually no higher than 0.1% (1 Al for every 1000 ATP) the avidity with which ATP binds Al(III) relative to its usual co-factor Mg(II) (*ca* 10^3^ times higher) means that when Al-ATP replaces Mg-ATP as a source of phosphate the subsequent activity of hexokinase will be significantly diminished. Under the same conditions but additionally in the presence of citrate (a significant competitor for Al(III) binding at pH ≥ 7) Al-ATP dissociates and the formation of Al-citrate complexes protects the enzyme–substrate complex from the inhibitory actions of Al-ATP. This account of the consequence of the contamination of commercial supplies of ATP by Al(III) is in itself an important lesson but it also has wider implications for the arrival of Al(III) in biota and specifically human beings. There are few more fundamental reactions in human biochemistry than the phosphorylation of glucose using ATP and it should be clear to all that such a reaction would not have been selected for in human biochemistry in the presence of biologically available Al(III) (5). The insidious advent of the aluminum age has been and will continue to be heralded by burgeoning examples of the biological availability of Al(III) and it is of critical importance that we both recognize this fact and, concomitantly, prepare ourselves to counter any role that Al(III) might be playing in human disease. Currently, political aspects of the use of aluminum are preventing a common sense approach to living in the aluminum age and complacency is ensuring an accelerated exposure to aluminum in our everyday lives and a burgeoning body burden of aluminum for each and every one of us. In extending the paradigm of ATP’s contamination by aluminum, one could say that laboratory Earth’s supplies of human beings are already significantly contaminated with aluminum and it is now an active participant in human evolution.

## Complacency and Aluminum

The most significant factor driving complacency about the potential dangers of aluminum is its omnipresence in modern life. Many will have attended scientific meetings where one of the “aluminum ambassadors” for the industry would give a presentation on how aluminum’s omnipresence was a sure sign of its essentiality. The logic goes that since it is present in every cell then it must be beneficial, we just do not know how it is beneficial. Today, we all have a body burden of aluminum and it is likely that it is present in every physical and chemical compartment in the human body. It is also true to say that it is burgeoning and tomorrow’s generation will have a higher body burden of aluminum (6). However, we have yet to identify a beneficial role for aluminum in any extant organism, never mind human beings, and so to extrapolate presence to essentiality without any mechanism may be more hopeful than realistic. Hopeful because the consequences of an ever increasing body burden of the biosphere’s most abundant ecotoxin and a known human neurotoxin can only be detrimental, at least in the shorter term. The argument will concern the degree of severity and not if aluminum is toxic. Another factor that also allows for a degree of complacency about human exposure to aluminum is the fact that aluminum is rarely acutely toxic in human beings. There have been instances of acute, fatal, intoxications by aluminum and most notably those known collectively as dialysis encephalopathy (7). However, in everyday life human beings are not exposed to levels of biologically available aluminum, which are responsible for immediate acute effects. I have described aluminum as a silent visitor to the human body perhaps only being noticed when specific toxicity thresholds are achieved. Indeed, a role for aluminum in such an instance may not be noticed either immediately or at all. If aluminum is not suspected as an etiological agent in a particular condition then it is quite unlikely that its role in the disease will be investigated.

## What are the Symptoms of Chronic Aluminum Intoxication in Human beings?

It is probably correct to assume that aluminum toxicity will not have a single motif or signature, which is specific to its actions. This means that in the absence of any suspicion of a role for aluminum, a physician is unlikely to (immediately) diagnose aluminum toxicity in affected individuals. A lack of symptoms, which are immediately recognizable as aluminum toxicity relates to the biological reactivity of ${\mathrm{Al}}_{\left(\mathrm{aq}\right)}^{3+}$ and its significant propensity to be bound by oxygen-based functional groups associated with myriad biomolecules (8). These reactions will include the substitution of aluminum for essential metals, the *de novo* binding of aluminum by non-specific binding sites on protein-based biomolecules, and cross-linking reactions involving biopolymers. Aluminum will also be bound by labile molecules in both intracellular and extracellular milieus and some of these interactions will involve its transportation as high- and low-molecular weight complexes throughout the body and, ultimately, the excretion of aluminum from the body (9). The potential for aluminum to interact with and to influence so many biochemical pathways means that the symptoms of its toxicity could be deficiency or sufficiency, agonistic or protagonistic, and any combination of these and other physiology-based events. The scientific literature does document links between aluminum exposure and human disease and I recently compiled a table of such conditions and these might be starting points in diagnosing possible aluminum toxicity (10). However, for aluminum to play a significant role in any disease-related event some degree of toxicity threshold must have been achieved. Essentially, the rate of delivery of ${\mathrm{Al}}_{\left(\mathrm{aq}\right)}^{3+}$ to target ligands must be sufficient to overcome the inherent robustness of systems that are under attack. In achieving this threshold either aluminum must accumulate over time within a particular compartment or possibly the administration of a single dose of aluminum could achieve such a threshold instantaneously. The latter is probably more unusual in human being’s everyday exposure to aluminum except, for example, where aluminum is administered as an adjuvant in vaccination and allergy immunotherapy (11, 12).

## How is Aluminum Getting into the Brain?

The blood–brain-barrier sets the brain apart from the other organs of the body. It is a selective barrier and its control over transport of substances into and out of the brain has resulted in the brain being populated by highly specialized post-mitotic cells or neurones. Whether the chicken or the egg came first the outcome is a unique organ of central importance to the functioning of most of the rest of the human body. The observation that aluminum enters the brain and accumulates with age reminds us that the brain must have evolved in the absence of biologically available aluminum and that only now with the advent of the aluminum age is the brain having to cope with a burgeoning burden of brain aluminum (13). Very little is understood as to how aluminum enters the brain and as to which of the possible mechanisms of entry are most significant (14). There will be movement of aluminum from the blood into the brain and so any activity, which results in aluminum entering the bloodstream, will add to its brain burden. The leakiness of the blood–brain-barrier to aluminum will be influenced by its physiology, for example, the degree of selectivity is expected to be lower in the fetus and neonate while it may also be adversely affected in certain disease states including aluminum-related disorders such as Alzheimer’s disease and multiple sclerosis. Indeed, aluminum is known to increase the leakiness of epithelial and endothelial barriers and in doing so could concomitantly increase the passage of aluminum from the blood to the brain (15). Blood to brain passage of aluminum is a constant pressure on the brain aluminum burden and its overall significance may depend upon an individual’s body burden of aluminum as well as their general health. Another route of entry of aluminum to the brain is the olfactory system and the movement of originally air-borne aluminum directly into the hippocampus (16). This method of uptake of aluminum into the brain becomes increasingly significant in individuals exposed to aerosols of particulate aluminum, for example, occupational exposure to aluminum dust (17). While the exposure is likely to be more sporadic than continuous exposure via the blood, it is also likely to be more acute with the strong possibility of significant uptake of aluminum over relatively shorter time periods, for example, years as opposed to decades of exposure. Aluminum might also enter the brain by diapedesis. For example, when aluminum adjuvants are used in vaccination and allergy immunotherapy immune cells, which infiltrate the injection site, are known to take up particulate aluminum adjuvant by endocytosis and translocate such throughout the body including into the brain via both paracellular and transcellular processes (18). The result is the potential delivery of significant quantities of adjuvant aluminum to the brain the fate of which remains to be elucidated.

## Why is the Brain a Likely Target Organ for Aluminum Toxicity in Human beings?

Neurones are the longest-lived cells of the human body and survive aging processes, which ravage the remainder of the human body (19). Evolution through natural selection has conferred biochemical advantages upon neurones and the neuronal microenvironment, which have in turn enabled human beings to live for longer. I would contend that the evolution of what is an ostensibly immortal cell line would not have occurred in the presence of biologically available aluminum. Indeed, the advent of the aluminum age must now have serious consequences for the health and longevity of such a cell line. The lifespan of neurones predisposes them to a lifetime accumulation of aluminum. Evidence for intraneuronal aluminum is incontrovertible though there is no consensus as to the mechanism of its uptake from the neuronal microenvironment (13). While a range of possible mechanisms are postulated none of these have been demonstrated for neurones and only endocytosis is confirmed for other cell types (20). The fate of intraneuronal aluminum is similarly obscure with evidence existing for its accumulation in nuclear compartments, including nucleoli, its deposition in lysosomes and other vesicle-based stores and its likely presence in a number of chemical compartments such as cytosolic pools of ATP and citrate (13). While neurones will accumulate aluminum it is also possible that some intraneuronal aluminum may be secreted from neuronal bodies as complexes with ligands such as ATP (21) and neurotransmitters, specifically glutamate (22). It is probable that the accumulation of aluminum in neurones accounts for a burgeoning brain burden of aluminum over lifetimes. Extraneuronal aluminum will move back into the periphery from the central nervous system either by piggy-backing on transport systems moving substances out of the brain or simply by residual leakiness and lymphatic drainage across the blood–brain-barrier. Thus, intraneuronal aluminum is both a sink for aluminum and a source of biologically reactive aluminum. The latter may be effective both at intraneuronal targets and, importantly, extraneuronal targets. What this means is that the potency of aluminum as a neurotoxin (concomitant with chronic lifetime exposure to aluminum) will be dictated by an intraneuronal threshold acting inside and/or outside the neuronal body to bring about wholly degenerative effects (23). The human brain is exposed to aluminum from the fetus to the grave. While the proportion of this aluminum, which is biologically available remains at a level, which can be endured without precipitating significant biological effects aluminum toxicity will not be manifested. However, at some point, which will be significantly influenced by individual circumstances, toxicity will be exerted, brain systems will become dysfunctional and cascades of events eventually leading to accelerated cell and neurone loss will begin to dominate. This is when aluminum becomes a neurotoxin (24).

## What are the Mechanisms of Neurotoxicity?

To appreciate the neurotoxic potential of aluminum is to recognize the myriad ways that biologically available aluminum can and will interfere with normal brain metabolism. Aluminum, primarily acting through ${\mathrm{Al}}_{\left(\mathrm{aq}\right)}^{3+},$ is a generalist and will affect multiple systems to bring about global changes influencing neuronal function and, ultimately survival. For example, biologically available aluminum through the potentiation of damaging redox activity (25) or the disruption of intracellular calcium signaling (26) will systematically wear down cellular defenses. The mechanism of toxicity of aluminum is invariably biphasic with lower concentrations producing toxic effects through stimulatory actions and higher concentrations, resulting in inhibition of essential processes and pathways (27). The omnipresence of a rogue metal ion, which is able to compete with and replace other metals in essential processes means that any chink in the armor of essential cellular systems will be exacerbated by the additional presence of aluminum. Aluminum kicks a process while it is down and potentially prevents it from getting back up again. The presence of aluminum in the brain must mean that it is inevitable that it will contribute toward any on-going degenerative conditions such as Alzheimer’s disease or multiple sclerosis, ultimately, resulting in earlier onset and/or more aggressive forms of the disease (17, 28). It is impossible with the data, which are currently available to be precise about mechanisms underlying aluminum’s role in neurodegenerative disease. It is probable that aluminum will affect early stages of diseases, for example, influencing gene expression (29), with concomitant effects upon functioning of neurones. However, it is unlikely that chronic exposure to aluminum will induce necrotic cell death and more likely to exert toxicity through stimulation and inducement of mechanisms of cell death on the continuum that includes such processes as autophagy and eventually apoptosis (30, 31). Aluminum helps to convince organelles and cells (neurones) that they are better off committing suicide than continuing to fight against their disruption and dysfunction. While the programed destruction of organelles and cells will involve their deconstruction to leave discrete packages for further metabolism the fate of aluminum in these processes is largely unknown though it is unlikely that it will leave the brain and some of it may be found in such structures as senile plaques, neurofibrillary tangles, Lewy bodies, and lipofuscin. All of which are examples or signatures (tombstones) of neurodegenerative disease (23).

## Solutions

The advent of the aluminum age and the consequence of the omnipresence of aluminum, not only in the environment but also throughout the human body, is that we are all subject to chronic aluminum intoxication. Every minute of each day we expend energy coping with the presence of biologically reactive aluminum in our bodies. The higher the body burden of aluminum the more likely that this coping mechanism will manifest itself as disease. In the brain, aluminum will contribute toward neurodegenerative diseases including Alzheimer’s disease, Parkinson’s disease, and multiple sclerosis. There is now a clear requirement for therapy or treatment, which could lower the body burden of aluminum, and particularly the aluminum content of the brain (32). It might, therefore, be surprising to find that there are no drugs or chelators, which have been developed and clinically approved for the specific purpose of removing aluminum from the body. The iron siderophore desferrioxamine (DFO) has been used in addressing aluminum overload, and successfully so in one trial of Alzheimer’s disease (33), and the general purpose metal chelator EDTA has some efficacy in facilitating the urinary excretion of aluminum (34). However, neither of these treatments is specific for aluminum and neither has been used with the stated aim of lowering the body burden of aluminum in healthy individuals. Surprising or otherwise there is a real and urgent need for treatments, which will facilitate the removal of aluminum from the body and preferably without disrupting essential metals, such as iron. The treatment should be as non-invasive as possible as it needs to be amenable to healthy individuals and individuals with aluminum-related conditions. It is equally important that the success of the treatment can be measured quantitatively, which means that we also need to fully understand the routes of excretion of aluminum from the body and the treatment’s impact upon these routes. We need reliable and reproducible measures of the body burden of aluminum in order that the impact of it being reduced can be related to any subsequent changes in health-related indices (6).

In the late 1980s, my Ph.D. research demonstrated the amelioration of acute aluminum toxicity in fish by silicon (35). The unique inorganic chemistry of the reaction of aluminum with silicic acid [Si(OH)_4_] the only biologically available form of silicon, has remained my life’s work and is now the subject of a possible therapy to facilitate the removal of aluminum from the human body (36). This therapy is based upon the observation that drinking silicon-rich mineral waters increases the excretion of aluminum in urine (37). Silicon in mineral water (and indeed most potable waters) is found mainly as silicic acid, which in the gut is immediately absorbed and enters the blood before being excreted in the urine via the kidney. Mirroring the urinary excretion of silicic acid is aluminum and this close relationship suggests that silicic acid in some way facilitates the excretion of aluminum via the kidney. The mechanism of action remains to be elucidated but it is thought to involve a pulse in silicic acid concentration in the blood, which facilitates the passage of low-molecular weight (<18 kDa) forms of aluminum across the glomeruli of the kidney. The facilitation might involve the formation of hydroxyaluminosilicates (HAS) as our most recent research on the speciation of aluminum in blood identified a non-equilibrium phase of aluminum hydroxide (38), which is a necessary precursor to the formation of HAS. While we have demonstrated this effect of silicon-rich mineral waters in healthy and in diseased individuals of all ages there remain a number of unresolved issues in relation to the efficacy of this treatment as a long-term therapy to reduce the body burden of aluminum. We need to establish if there is a lower limit for the silicon content below which there is no concomitant removal of aluminum from the body. At present, we have set this limit at 30 mg/L “silica” (as usually written on bottles) or 14 mg/L (0.5 mM) as silicon (silicic acid). We also need to understand the volume of water, which should be drunk each day and whether or not this volume should be taken as many small aliquots or several large aliquots. Our default position at the moment is that a minimum of 1 L should be drunk each day and it should be taken as only a few aliquots. While the majority of individuals who begin to drink the mineral water, as recommended above, show immediate increases in their urinary excretion of aluminum we do not have excretion data beyond 7 weeks for healthy volunteers and we do not know how long it will take for excretion data to indicate statistically significant reductions in individual’s body burdens of aluminum. While it is important that we obtain such data our default position on this is that to achieve maximum protection against everyday human exposure to aluminum the consumption of a silicon-rich mineral water should become a normal part of an individual’s diet and lifestyle. In a small cohort of individuals with Alzheimer’s disease, we were able to demonstrate a statistically significant reduction in their body burdens of aluminum over 12 weeks of treatment while in their age and gender-matched control population the reductions in body burden of aluminum did not reach statistical significance in the same period (39). We do not, of course, know if aluminum is being removed from all of the body, for example, if it is being purged from the brain. The assumption, which is as good as we can make at the moment, is that all body stores of aluminum will be in some sort of dynamic equilibrium with the blood and so the removal of aluminum from the blood via the kidney will drive the removal of aluminum from other tissues including the brain. Tentative support for the removal of aluminum from the brain comes from our recent study on Alzheimer’s disease where 3 out of 15 individuals with the disease showed clinically relevant improvements in cognitive performance by the end of the study (39). Up until very recently, we assumed that the major route of elimination of systemic aluminum was in urine. However, our recent observation of high concentrations of aluminum in perspiration (40) may require a modification of this assumption and will also require better understanding of whether silicon-rich mineral waters might also facilitate the removal of aluminum from the body in perspiration.

## Future Perspectives

We are living in an aluminum age and it is highly probable that our use of aluminum will increase in the future (6). The parallel recent histories of acid rain and intensive agriculture are immediate testimonies to the ecotoxicity of a burgeoning biotic burden of aluminum and it is inevitable that our increasing exposure to aluminum will (continue to) impact upon human health. There has been and there continues to be systematic attempts by the aluminum industry to suppress research on aluminum and human health. While independent research in this field is prevented the questions concerning human toxicity remain unanswered. Lack of required research does not equate to lack of biological effect or safety and neither does it implicate aluminum as a cause of human disease. However, while our everyday exposure to aluminum may not be the cause of any number of chronic human conditions it most certainly can be and probably is a contributor to such diseases. The evolution of modern human beings began in the absence of biologically available aluminum and is now progressing in the presence of a burgeoning body burden of what we know to be the most ubiquitous and abundant ecotoxin on Earth. The aluminum age is here to stay and it is now the responsibility of those organizations charged with protecting the health of nations to introduce legislation to limit human exposure to aluminum and so to ensure that we can live safely and effectively alongside the World’s favorite metal.

## Conflict of Interest Statement

The author declares that the research was conducted in the absence of any commercial or financial relationships that could be construed as a potential conflict of interest.
